# Is Cognitive Change Necessary to Alleviate Symptoms in Patients With Functional Somatic Syndrome?

**DOI:** 10.3389/fpsyt.2021.781083

**Published:** 2021-11-02

**Authors:** Daniel Maroti, Robert Johansson

**Affiliations:** ^1^Department of Clinical Neuroscience, Karolinska Institutet, Stockholm, Sweden; ^2^Department of Psychology, Stockholm University, Stockholm, Sweden

**Keywords:** functional somatic syndrome, IBS, fibromyalgia, chronic fatigue syndrome, reattribution, dysfunctional belief, cognitive restructuring

## Introduction

What has been included under the umbrella term “Functional somatic syndromes” (FSS) has varied over the years. Diagnoses such as “somatoform” and “somatic symptom disorders,” as well as “medically unexplained symptoms,” have been included in FSS (1). Although there is no complete consensus, FSS usually comprise of conditions such as fibromyalgia, irritable bowel syndrome (IBS), and chronic fatigue syndrome (CFS) (2). A characteristic of FSS is persistent physical symptoms that lead to impairment or disability and that disrupt the capacity to take part in daily life. Although FSS are believed to be caused by a complex interaction of biological and psychosocial factors (3), the conditions are not medically explained in terms of well-Understood or established pathophysiological mechanisms.

Cognitive behavioral therapy (CBT) has become the gold standard psychological treatment for FSS. Several meta-analyses have shown a small to moderate effect in patients with FSS (4–7). Even though the small to moderate effect is robust, in the sense that results have been replicated both within and across conditions, there is limited research on treatment mechanisms (8). That is, CBT has an effect, but why the effect takes place is not firmly established.

Traditional CBT rests on the idea that changing cognitions and behaviors in FSS will decrease somatic symptoms (9). Specifically, changing so-called *dysfunctional illness beliefs* “are of paramount importance for treatment (success)” (10). According to a review of Windgassen et al. (11), there is a “clear indication” that “cognitive change is important for reducing symptom severity.”

Put simply, a patient who, for example, believes that “something is wrong with my body” or claims a physical attribution such as “I have a chronic infection, that is why I feel fatigued,” will not easily get well. There are some merits to this position. In a review of CBT for IBS, four out of five studies found cognitions to mediate the effect of treatment on symptom severity (11). In another review of CBT for CFS, three different types of illness perceptions (fatigue as something aversive, activity as potentially dangerous to health, and a symptom preoccupation with fatigue) were found to potentially perpetuate CFS symptoms (12).

Although there is a value in the position that changing cognition is of “paramount importance” to reduce symptoms in FSS, we argue that this idea is a simplification and leaves several questions unanswered. In the following, we provide two major arguments against this position.

### Counterargument 1: Patients With FSS Hold Complex Illness Beliefs or Attributions

Patients with FSS have been assumed to hold strong beliefs that the cause of their symptoms is a physical disease (10). It has been suggested that they inflexibly hold assumptions like “something is wrong with my body” or “there is nothing I can do except waiting for a medical cure.” While it might be true that there is a tendency among certain patients with FSS to hold such beliefs (13, 14), some research speaks to the fact that patients with FSS either do not hold clear causal attributions or have a plethora of quite complex illness attributions.

For example, in a study of 104 patients with fibromyalgia, over two-thirds of the patients endorsed the statement that “I cannot understand at all the cause of my pain (the reason I have pain)” (15). In these patients, there simply are no dysfunctional illness beliefs to change, as they do not understand the reason for why they have pain.

In a study of 66 patients with chronic fatigue syndrome, half of the sample believed that stress had played a role in the development of their illness (16). In another qualitative study based on nine adolescents with CFS, three out of nine participants had a multi-causal understanding of their illness, believing that the illness had both physical and psychological origins (17). It is not clear how a patient who holds a psychological attribution fits with the idea of changing dysfunctional illness beliefs about the illness as a pervasive and uncontrollable physical disease.

### Counterargument 2: Patients Can Get Better Even if There Is No Change in Illness Attributions

If dysfunctional illness beliefs are necessary to reduce somatic symptoms, people could not get better if the illness beliefs are left unaltered. However, this does not seem to be the case. In a randomized controlled trial of CBT for CFS, CBT was found to be superior to a control condition of relaxation training and 70% of those who completed CBT achieved a good outcome (18). However, physical illness attributions, which were widespread, did not change in response to treatment and were not predictive of improved outcomes. If people can get better without changing their illness attribution, other mechanisms are likely responsible for this improvement.

Furthermore, in a rigorously conducted study of mediators in patients with fibromyalgia (19), three mediators were related to a successful treatment outcome: (1) avoidance behavior, (2) mindful non-Reactivity, and (3) cognitions/worry (holding catastrophizing beliefs such as “exercise will make me sick”). However, when a time-lagged analysis was conducted, it was only the prevention of avoidance behavior that acted like a probable causal agent in reducing pain. That is, although cognitions were related to treatment outcome, dysfunctional illness beliefs were not a necessary condition for change.

## Discussion

In this article, we have argued that while CBT produces a robust small to moderate effect in FSS, it is less clear if altered dysfunctional illness beliefs form the most important vehicle of change in *all* patients with FSS or to *all* improvements in symptoms in patients with FSS. We have provided two major arguments for why altered dysfunctional illness beliefs might not be of “paramount importance” for treatment success. They could be summarized as (1) patients with FSS hold complex illness beliefs or attributions and not simply dysfunctional illness beliefs, (2) patients can get better even if there is no change in illness attributions. These two arguments highlight difficulties with the idea that changing dysfunctional illness beliefs is necessary for patients with FSS to reduce somatic symptoms. Arguments such as these have previously been raised by other authors (20).

To be fair, some of the CBT models of, for example, CFS do not explicitly state that cognitions are the causative agent of fatigue. Instead, cognitions are believed to be related to behavior, which in turn influences somatic symptoms (21). Nevertheless, some proponents of CBT still stress that cognitions should be the target of change in treatment (22).

Most researchers would argue that various mechanisms, and not just cognitions, are important in the treatment of FSS. One is, as previously discussed, behavioral avoidance (19). Another potential mechanism is that of emotional processing. In a study of Emotional Awareness and Expression Therapy for FSS patients, a mediation analysis distinguished two emotional processes that were associated with reductions of somatic symptoms (23). One was “impoverished emotional experience,” which is similar to the concept of alexithymia. The other process was “signs of unprocessed emotions,” where memories and emotions feel fragmented and intrusive. The mediation analysis showed an association between the ability to identify and make better sense of emotions without finding them intrusive and a reduction in somatic symptoms. This research suggests that there are other possible mechanisms of change in the treatment of FSS besides cognitions.

As FSS are heterogeneous disorders, a “one size fits all” approach is not the way forward. Changing dysfunctional beliefs through CBT may be important in some patients with FSS but not to others because of the different characteristics and risk factors (24, 25).

Future treatment studies of FSS should be informed by theory and include the important mediating variables that are hypothesized to change with treatment and in turn influence the outcome. To establish which mediating variables are of importance, two different treatment protocols with their own distinctive mechanism would ideally be compared.

## Author Contributions

DM conceived the manuscript and revised the drafts, and wrote the first draft. RJ contributed with improvements of the manuscript. All authors contributed to the article and approved the submitted version.

## Funding

This study was funded by foundation of the Söderström König Foundation and Karolinska Institute's Research Foundation Grants.

## Conflict of Interest

The authors declare that the research was conducted in the absence of any commercial or financial relationships that could be construed as a potential conflict of interest.

## Publisher's Note

All claims expressed in this article are solely those of the authors and do not necessarily represent those of their affiliated organizations, or those of the publisher, the editors and the reviewers. Any product that may be evaluated in this article, or claim that may be made by its manufacturer, is not guaranteed or endorsed by the publisher.
